# Delineation of somatosensory finger areas using vibrotactile stimulation, an ECoG study

**DOI:** 10.1002/brb3.369

**Published:** 2015-09-20

**Authors:** Rémy Wahnoun, Michelle Benson, Stephen Helms‐Tillery, P. David Adelson

**Affiliations:** ^1^ Barrow Neurological Institute at Phoenix Children's HospitalChildren's Neuroscience ResearchPhoenixArizona; ^2^ School of Biological and Health Systems EngineeringArizona State UniversityTempeArizona

**Keywords:** Electroencephalography, epilepsy, event‐related potentials, evoked potentials, fingers, somatosensory cortex

## Abstract

**Background:**

In surgical planning for epileptic focus resection, functional mapping of eloquent cortex is attained through direct electrical stimulation of the brain. This procedure is uncomfortable, can trigger seizures or nausea, and relies on subjective evaluation. We hypothesize that a method combining vibrotactile stimulation and statistical clustering may provide improved somatosensory mapping.

**Methods:**

Seven pediatric candidates for surgical resection underwent a task in which their fingers were independently stimulated using a custom designed finger pad, during electrocorticographic monitoring. A cluster‐based statistical analysis was then performed to localize the elicited activity on the recording grids.

**Results:**

Mid‐Gamma clusters (65–115 Hz) arose in areas consistent with anatomical predictions as well as clinical findings, with five subjects presenting a somatotopic organization of the fingers. This process allowed us to delineate finger representation even in patients who were sleeping, with strong interictal activity, or when electrical stimulation did not successfully locate eloquent areas.

**Conclusions:**

We suggest that this scheme, relying on the endogenous neural response rather than exogenous electrical activation, could eventually be extended to map other sensory areas and provide a faster and more objective map to better anticipate outcomes of surgical resection.

## Introduction

### Epilepsy monitoring

Approximately 65 million individuals worldwide are living with epilepsy, 2.2 million of whom are in the United States (Epilepsy Foundation, 2013). The first and most common form of relief relies on anti‐epileptic drugs. However, one‐fourth to one‐third of the cases do not become seizure free from drug therapy alone (Téllez‐Zenteno et al. 2005; Privitera 2011). In these situations, surgery may be an option if a single, localizable focus can be identified and safely removed. Generalized seizures, arising within and rapidly engaging bilaterally distributed networks, or seizures localized in the language areas of the brain may not be resectable and therefore surgical strategies would more likely entail disconnection to interrupt seizure spread through the network or alternatively, neuromodulation.

To obtain a broad sense of the origins and types of seizures, neural activity is first monitored using an electroencephalography (EEG) system through scalp recordings of brain activity (Phase I). If the seizures appear to be potentially focal and unilateral, surgically implanted electrocorticographic (ECoG) electrodes on the cortical surface, or depth electrodes for deep foci, are used to monitor cortical activity during seizures and further define the epileptic foci (Phase II). The decision for surgical resection or intervention depends on the data from this invasive monitoring strategy clearly delineating the epileptogenic zone and ensuring that resection of the seizure foci will not significantly impact neurological functions. During Phase II monitoring, in addition to seizure localization, several procedures are us*e*d to define areas of eloquent cortex and attempt to estimate the cognitive functions possibly affected by respective surgery.

### Standard sensorimotor mapping

Electrical cortical stimulation (ECS) is considered the gold standard for sensorimotor functional delineation of eloquent tissue in the brain. In contrast to continuous monitoring where the electrical current from the brain is passively recorded, electrical current is passed between neighboring electrodes to evoke sensory or motor manifestations. Typically during ECS, 50 Hz square pulse trains are applied lasting two to 5 sec (Ikeda et al. 2002). The stimulation current is gradually increased up to 10 mA until a sensory, a motor, or an after‐discharge response is elicited.

A bottom‐up approach can also be performed by electrically stimulating peripheral nerves and visually observing evoked responses in the cortical signals. These methods result in the construction of a somatotopic map of sensory and motor function.

However, those two techniques have limitations. The somatic response is subjective and interpretative based on the patient's response and direct observation by the tester. For sensory areas, it is often difficult to interpret evoked stimuli. In children, particularly those who are too young or nonverbal due to cognitive dysfunction, interpretation of sensation can be very difficult. Additionally, after‐discharges, an unwanted consequence of ECS stimulation, are frequent, and can lead to seizures. Unfortunately, stimulation‐evoked seizures have poor diagnostic value as they do not show a strong correlation with natural seizure foci (Blume et al. 2004).

Cortical stimulation does not always elicit motor responses in children under ten years of age (Haseeb et al. 2007; Connolly et al. 2010). In addition, sensory mapping often relies on the patient's ability to describe sensations or follow directions, which is often dramatically lowered as the patients are recovering from the ECoG implantation during the invasive monitoring period. Cortical mapping can be uncomfortable and trigger nausea or seizures which at best can prolong the process considerably, and at worst it can result in the postponement of the clinical mapping (Selimbeyoglu and Parvizi 2010).

The present study evaluates a potential alternative approach, involving vibratory stimulation of individual fingers and a cluster‐based analysis in the time‐frequency domain, to avoid the deficiencies listed above.

### Somatosensory pathway

Vibratory stimuli, such as those used in our protocol, are relayed by the lemniscal pathway from the cutaneous mechanoreceptors to the somatosensory cortical areas (Patestas and Gartner 2006; Cruccu et al. 2008). Previous somatosensory evoked magnetic fields studies on healthy subjects suggest that epicritic inputs from the lemniscal system are transmitted from the ventroposteriolateral nucleus of the thalamus to several cortical areas; Information is thought to be transformed in a hierarchical way from area 3b, in the posterior wall of the central sulcus to areas 1 and 2 on the surface and area SII in the upper bank of the Sylvian fissure (Inui et al. 2004; Kalberlah et al. 2013). After pneumatic activation of mechanoreceptors, a strong response is first observed in the contralateral SI, followed by a bilateral response in SII (Simões et al. 2001). In addition to lemniscal inputs, multimodal studies demonstrate that inputs conveying heat and pain and transmitted separately in the spinothalamic system (Dijkerman and de Haan 2007; Liang et al. 2011).

Finger representation in the contralateral SI covers a 10–20 mm long cortical strip (Penfield and Boldrey 1937; Pollok et al. 2002; Overduin and Servos 2004), following a latero‐medial distribution, from the thumb to the small finger with a limited amount of overlap (Simões et al. 2001; Schweizer et al. 2008), and notable inter‐individual variability (Schweizer et al. 2008). The volume of cortical representation of the digits shows some relative correlation to the receptor density of the fingers, and is larger for the thumb than the index, and ring fingers (Overduin and Servos 2004). Electrical, vibrotactile and mechanical stimulation studies suggest that SII does not seem to follow a topological organization of the fingers (Kalberlah et al. 2013) or show a strong spatial overlap (Ruben et al. 2001; Simões et al. 2001), and may be involved in bimanual tasks (Simões et al. 2001).

### Vibrotactile mapping

In fMRI and EEG studies, vibrotactile stimulation was successfully used to study somatosensory areas (see Table 1). The slow and fast frequencies in these studies are thought to mainly activate respectively the Meissner (20–50 Hz) and Pacinian (60–400 Hz) corpuscles (Kandel et al. 2000). In this study 200 Hz vibrations are used for stimulation, near the peak response of the Pacinian corpuscles.

**Table 1:** Examples of previous vibrotactile mapping

Stimulator	Frequencies	Aim	Study
Piezo‐electric braille display	30 Hz, 200 Hz	S1 S2 relationship	Kalberlah et al. (2013)
Electromechanical vibrator	24 Hz, 240 Hz	S1 S2 relationship	Hämäläinen et al. (1990)
Compressed air driven off center mass	40–50 Hz	Locate S1, S2 sensory thalamus	Chakravarty et al. (2009)
Piezo‐ceramic stimulator	15 Hz, 30 Hz	Individual fingers delineation	Maldjian et al. (1999)
Braille piezo‐stimulator	16 Hz	Individual fingers delineation	Schweizer et al. (2008)

### Time domain analysis

Time domain analysis typically focuses on evoked potentials, averaging cortical responses over large numbers of trials. This procedure enhances time locked components and reduces the impact of nonrelated activity. Somatosensory Evoked Potentials (SEPs) can be elicited by stimulating peripheral nerve fibers (Allison 1987) and are conventionally named after their polarity followed by their latency in milliseconds.

In an MEG‐based study Simões et al. (2001) located three current dipoles elicited by air stimulation of the fingers; A P66 in contralateral SI, followed by a P100 in contralateral SII, and P111 in ipsilateral SII.

In the post‐Rolandic contralateral EEG, notable somatosensory responses to short tactile pulses consist of the sequence P50, N70, P100, N140, then P300 (Hämäläinen et al. 1990; Eimer and Forster 2003). N140 is largest in the contralateral cortex and often presents a bifid peak shape. Vibratory stimulation exhibits similar responses, but with a larger P100 as compared to tactile pulses (Hämäläinen et al. 1990).

### Time‐frequency domain analysis

Time‐frequency domain analysis expands the analysis of evoked responses in terms of changes in oscillatory activity induced by stimulation. Evoked Response Synchronization (ERS) and Evoked Response Desynchronization (ERD) correspond respectively to an increase or decrease of the power of oscillations in a given band. The general assumption is that ERS emerges as the result of a surge of concurrent activity in a network, while ERD arises as the result of a decreased correlation (Neuper and Pfurtscheller 2001). It has also been hypothesized that smaller functional networks may exhibit higher ERS frequencies than larger areas (Singer 1993; Pfurtscheller et al. 2001).

Somatosensory stimulation has been shown to result in an increase of oscillatory activity in the Gamma range, as well as a decrease in Alpha and Beta bands in the contralateral postcentral cortex (Pfurtscheller et al. 2001; Fukuda et al. 2008, 2010), with hints that the initial processing of the stimuli may initiate in the high‐frequency domain (Fukuda et al. 2010). Some of these induced oscillations are phase locked to the stimulus and appear concurrently with SEP components. Both phase‐locked and nonphase‐locked oscillations are believed to be present in sensorimotor studies (Fukuda et al. 2010).

During movement preparation, a contralateral ERD (below 30 Hz) is followed by bilateral ERD and associated contralateral ERS (above 30 Hz) during execution. Finally, around 700 msec after movement onset, a Beta resynchronization signals a return to baseline (Salmelin et al. 1995; Pfurtscheller and Lopes da Silva 1999; Pfurtscheller et al. 2001, 2003).

In this paper, we propose an objective method for defining finger‐related areas that does not depend on biased interpretation and does not involve direct cortical stimulation. This method produces maps even in the case of unresponsive patients. We suggest that this scheme can prove more effective and less distressing to patients in defining eloquent areas of cerebral cortex.

## Materials and Methods

### Subjects

Twelve patients undergoing Phase II monitoring for epileptic focus resection at Barrow Neurological Institute at Phoenix Children's Hospital participated in this study. The scope of this paper was restricted to subjects (three males. four females, mean age: 11.6 years, range: 5–20 years) for whom either the clinical or the vibrotactile mapping identified finger or hand responses (see Table 2). The remaining subjects were excluded because their ECoG grids were outside the area of interest (two subjects), because clinicians were unable to obtain clinical sensorimotor maps results (two subjects), or because of incomplete recordings (one subject).

**Table 2:** Demographics

	Gender	Age (years)	Tasks				Wakefulness	Etiology	Surgical alleviation
			CLs	ILs	CLsm	ILsm			
Sub1	M	11.8	Yes	Yes	Yes	Yes	Awake	Tuberous sclerosis	Multiple resection
Sub2	F	5.5	Yes	Yes	No	No	Awake	Developmental impairment	Local resection
Sub3	F	8.3	Yes	Yes	No	No	Sleeping	Cortical dysplasia	Local resection
Sub4	F	8.7	Yes	Yes	No	No	Awake	Encephalopathy	None
Sub5	M	10.9	Yes	Yes	Yes	Yes	Awake	Lennox Gastaut syndrome	VNS placement
Sub6	F	20.1	Yes	Yes	Yes	Yes	Awake	Cortical dysplasia	Temporal lobectomy
Sub7	M	15.8	Yes	Yes	Yes	Yes	Awake	Perinatal depression with intracranial hemorrhage	Occipital parietal lobectomy

All described procedures were approved by Phoenix Children's Hospital Institutional Review Board and written informed consent was obtained by the parents or legal guardians and/or the subjects prior to any procedure.

Grid locations were selected from the observations from Phase I monitoring and no additional implants were placed for this study. Sub3 was asleep during all the experimental recordings.

### Data acquisition

Patients were implanted with titanium ECoG electrode grids (10 mm inter‐contact distance, 4 mm diameter) and/or strips manufactured by Ad‐Tech Medical Instrument Corporation (Hartland, WI) or Integra LifeSciences (Plainsboro, NJ) according to clinical needs. Reference and ground electrodes were placed in a distant location on either contralateral or ipsilateral cortex, according to patient‐specific surgical accessibility.

In contrast to EEG recordings, ECoG electrodes lie on the surface of the brain, offering increased spatial resolution and higher signal to noise ratio. A Ripple Grapevine amplifier (Salt Lake City, UT) was used to record the cortical signals at 500 Hz concurrently with the standard clinical setup (Xl‐Tek EMU 128). The software Bci2000 (Schalk et al. 2004) orchestrated stimulation and data acquisition. An Integra OCS2 Ojemann Cortical Stimulator Integra LifeSciences was used for the clinical mapping. Three‐dimensional models of the patients’ brains, grids, and landmark locations were obtained using CT and MR scans. The Freesurfer image analysis suite was used for volumetric segmentation and cortical reconstruction (Reuter et al. 2010, 2012) from MRI images. Grid extraction from CT scans and co‐registration with MR scans were achieved with 3D Slicer 4.3 (Fedorov et al. 2012). Tridimensional models and analysis results were rendered using Blender 2.73 (Stichting Blender Foundation, Amsterdam, the Netherlands). Due to postoperative swelling and the integration of preoperative MRI with postoperative CT, the recording grids were realigned radially to match the pial surface. Due to an inferior MRI quality on one subject, the tridimensional model with the closest structural match was used for this subject.

### Vibrotactile stimulator

**Figure 1:**
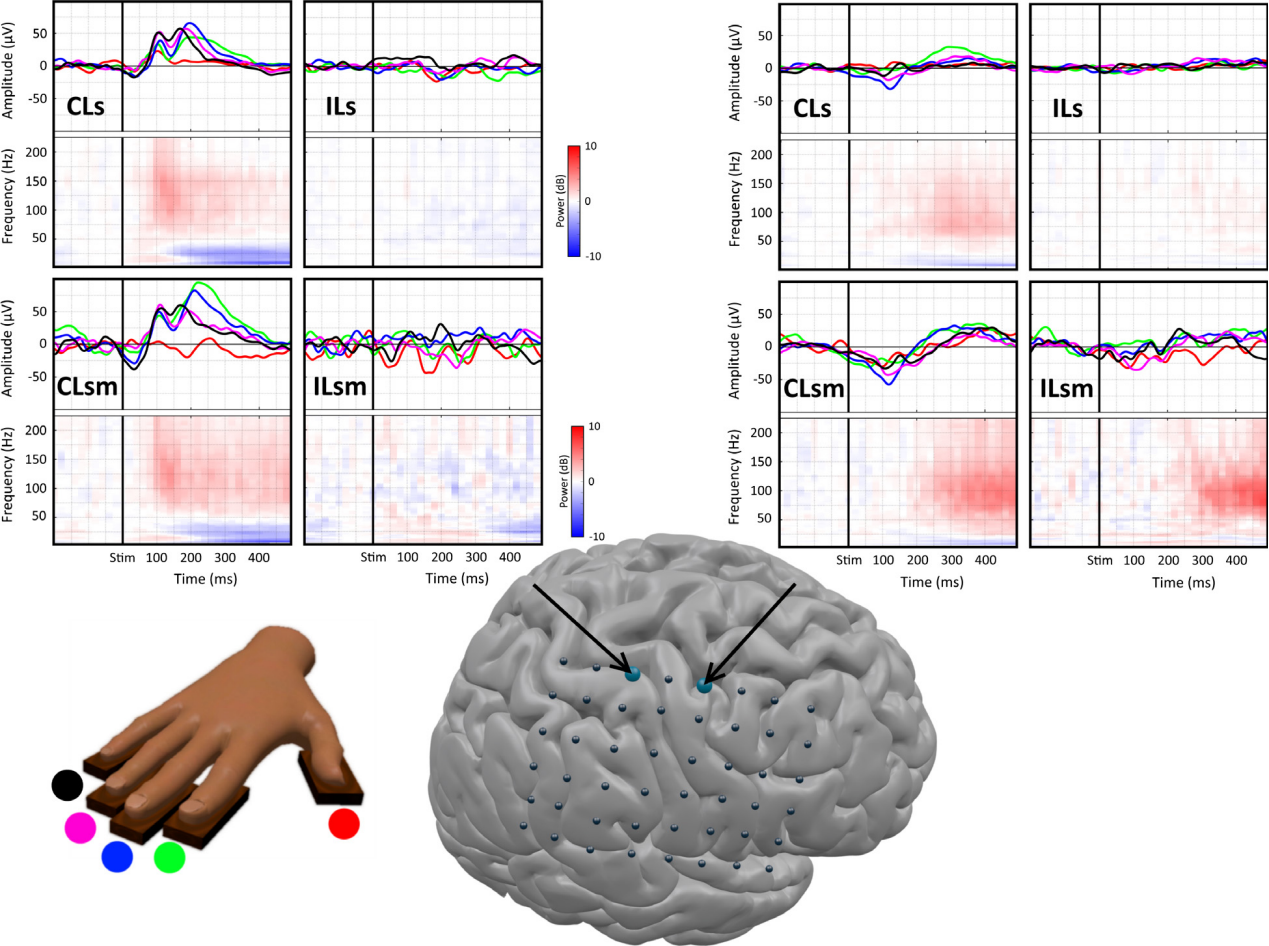
Temporal and Spectrotemporal response to vibrotactile stimuli on Sub1. The two panels represent the average response to vibrotactile stimulation under the four experimental conditions (CLs, contralateral sensory; CLsm, contralateral sensorimotor; ILs, ipsilateral sensory; and ILsm, ipsilateral sensorimotor). The top of each subpanel represents the average evoked response; the bottom part shows its average time‐frequency representation over the five fingers (ERS in red, ERD in blue). Traces are color‐coded per stimulated finger as shown in the bottom‐left sketch.

We designed a finger stimulator (see Fig. 1), consisting of five 10 mm shaftless vibration motors (Precision Microdrives, Vibration frequency: 200 Hz, Precision Microdrives Ltd, Lond.) and five 0.5″ force sensitive resistors (Interlink Electronics, Camarillo, CA), each encased in custom 3D‐printed finger placeholders. The individual units were placed on a slotted foam pad, allowing for adjustment to the size variations in our young population, and reducing vibration interference between the fingers. The system was controlled by an Arduino Pro Micro and connected to a computer via USB, allowing bidirectional communication with the recording computer.

A custom made shield was designed to provide sufficient current to the motors and optically isolate the patients from the computer. Bci2000's source code was then modified to interact with the stimulator, triggering vibrations and synchronizing pressure values from the five fingers to the cortical signals. We measured the delay as well as rising time of the motors for correction in the data analysis.

### Tasks

Subjects performed two tasks: a sensory only (s) and a sensorimotor (sm) task, performed contralaterally (CL) or ipsilaterally (IL) to the recording grids of interest, leading to four experimental conditions: CLs, CLsm, ILs, and ILsm.

In the sensory only task, while patients sat comfortably in their hospital bed, we placed their fingers on a stimulation pad designed to individually stimulate the fingers. One hand at a time, the patients’ fingers were randomly vibrated for a period ranging from 100 msec to 500 msec, with an inter‐stimulation interval ranging from 1 sec to 1.4 sec.

In the sensorimotor task, the stimulation remained the same, but the patients were instructed to attend to the stimulus by pressing the corresponding finger on the recording pad. The time allowed for motor response was set to 4 sec, with an inter‐trial interval of 1.5 sec. Each set consisted of 50 trials (ten per finger), after which we switched hands. We recorded up to 30 sets per subject over a period of several days according to the each individual's physical and mental status.

### Data analysis

#### Preprocessing

Data processing and analysis were performed under Matlab 2014b (RRID:nlx_153890), using the FieldTrip (RRID:nlx_143928) toolbox (Oostenveld et al. 2011). The Bci2000 sets were imported into Matlab, baseline corrected, and segmented into 2 sec trials centered on stimulation onset. For grids showing significant common noise, individual channels were re‐referenced using a common average reference, on a per‐grid basis. The resulting trials were then band‐pass filtered (two‐pass windowed linear‐phase FIR) prior to time‐only (BP: 1–30 Hz) and time‐frequency analysis (BP: 1–230 Hz). A shorter trial interval (−150 to 500 msec) was then used for data analysis to reduce boundary effects after filtering and frequency analysis. Time‐frequency representations were calculated using a Morlet wavelet transformation with multiplication in the frequency domain (20 msec windows, 1 Hz precision, seven cycles).

Background cortical recordings show a significant amount of natural fluctuations due to variations in arousal, focus, or uncontrolled external stimuli during the course of the experiment, all of which could affect the experimental outcome. To minimize this effect, we recorded as many trials as possible for each condition and for all statistical tests; baseline correction was performed from 150 msec to 10 msec before stimulation. For time series, the average value over the baseline period was removed from each trial. For time‐frequency series, the trial power of each frequency was divided by its baseline value, then subject to a natural logarithmic transformation, resulting in values in decibels.

### Statistical analysis

In this study, we aimed to test for a potential effect of vibrotactile stimulation and locate this effect in the time‐frequency domain. A standard way to minimize the multiple comparison problem (arising when performing simultaneously a large amount of statistical tests) involves the Bonferonni correction. This is a very conservative correction for the large amounts of time‐frequency‐samples that we aim to simultaneously test, and little or no sample pairs would show significant effects.

To address non‐Gaussian distributions and multiple comparisons issues, we used a cluster‐based nonparametric permutation test described in (Maris and Oostenveld 2007). This test takes advantage of the fact that there is a significant correlation between adjacent time/frequencies/electrodes to lower the FWER, while reaching sensitivity above the Bonferonni correction.

This method results in clusters of adjacent time‐space or time‐frequency‐space samples with significant *P*‐values. We used the maximum *P*‐value of each cluster and ran 1000 Monte Carlo random partitions to calculate significance probability. We analyzed the responses separately, on a per band basis, defined as Alpha (8–13 Hz), Beta (13–30 Hz), LowG (30–55 Hz), MidG (65–115 Hz), and HigG (125–230 Hz).

## Results

### Examples of cortical response patterns to vibratory stimulation

This section describes the results obtained with Sub1 across all four experimental conditions.

For this subject, evoked responses are shown in the time domain (traces color‐coded by finger) and in the time‐frequency domain (Fig. 1) after contralateral vibratory stimulation. The first significant peak in the postcentral‐medial (Fig. 1, left) electrode is a long‐latency N40 peak across fingers at an average at 38 msec, followed by P100 (108 msec), N140 (138 msec), and P200 (186 msec), before returning to baseline levels. Short latency responses to vibratory stimulation were weak and disappeared after signal processing, which is consistent with previous studies (Feinsod et al. 1973; Hämäläinen et al. 1990).

The frequency domain response shows a wide broadband increase in the high‐frequency range coinciding with the first peak, followed by a low‐frequency rebound. Similar responses for this electrode can be seen in the contralateral sensorimotor task, with larger amplitudes.

The precentral‐medial electrode (Fig. 1, Right) presents a N100 followed by a high‐frequency increase in both contralateral tasks, as well as a delayed high‐frequency increase in the ILsm task.

**Figure 2:**
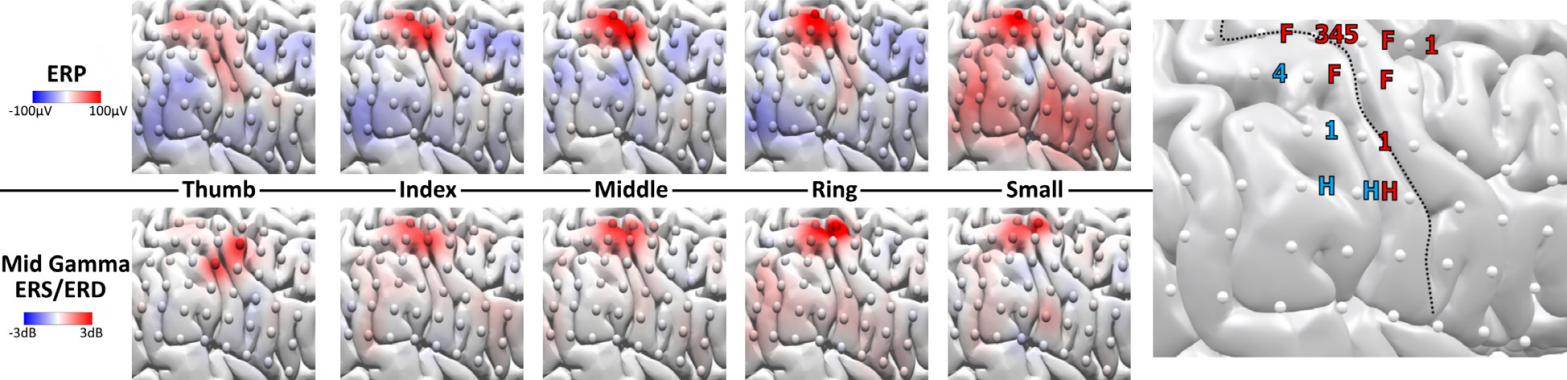
Sub1, Elicited response (80–120 msec) in the temporal (Left‐top), and spectrotemporal (Left‐bottom) domains to vibratory contralateral stimulation of the five fingers, and clinical electrical mapping (Right). Labels represent clinical mapping results (H, hand; F, fingers, numbers represent individual digits, s/m respectively sensory and motor).

Across subjects, the time‐frequency domain showed more spatially localized responses, as shown for Sub1 in Figure 2 (Left). For this subject, both domains presented the accepted precentral latero‐medial somatotopic distribution. However, in the frequency domain, the elicited response is more demarcated from the background and more spatially focused. For this subject, clinical mapping (Fig. 2, Right) located broad fingers/hand regions, but in some cases, postcentral stimulation lead to motor responses.

### Clustering results in the time‐frequency domain

Across all subjects, conditions and stimulated fingers, clustering in the spectrotemporal domain led to 151 significant (*P* ≤ 0.05) clusters appearing within 240 msec after stimulation (Table 3); 40.4% of which started as contralateral ERS above 65 Hz, while 17.9% corresponded to a contralateral ERD below 30 Hz.

**Table 3:** Number of significant ERS/ERD clusters across conditions and subjects (*P* ≤ 0.05)

	Alpha	Beta	LowG	MidG	HigG
CLs	2/8	0/13	0/2	29/1	11/1
CLsm	3/1	1/5	1/1	14/1	7/0
ILs	4/2	3/2	3/1	4/3	10/2
ILsm	4/1	1/1	1/2	2/2	1/1

ERS, evoked response synchronization; ERD, evoked response desynchronization; CLs, contralaterally sensory; CLsm, contralaterally sensorimotor; ILs, ipsilaterally sensory; ILsm, ipsilaterally sensorimotor.

The smaller number seen in CLsm as compared to CLs seems to originate from a higher residual prestimulus activity, preventing some clusters to exhibit significance at this level.

Significant clusters emerged earlier in the most active ERD band (MidG: 118.1 msec) than in the most active ERD band (Beta: 143.4 msec).

**Figure 3:**
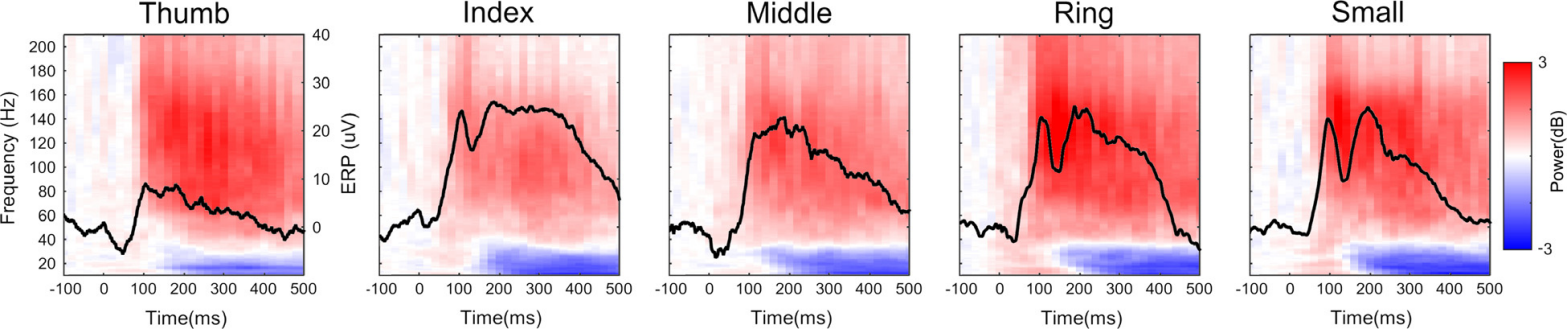
Time‐frequency evolution of electrodes with significant Mid‐Gamma clusters, and associated Event‐Related Potentials (black traces), averaged across subjects.

Due to the latency jitter often observed in the time domain and the inherent high background noise in our patient population, we focused in this study on the detection in the time‐frequency domain, and specifically the Mid‐Gamma event‐related synchronization. The criteria used for selecting this band were the high number of clusters and early activation. Across subjects, the electrodes presenting significant clusters in the MidG band presented an early ERS (red) followed by a late ERD (blue), as confirmed by Figure 3.

### Comparison with clinical mapping

**Figure 4:**
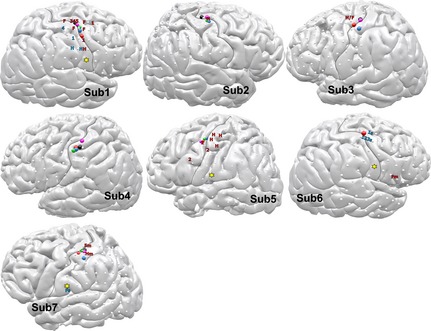
Clinical electrical mapping versus vibrotactile sensory mapping of the contralateral fingers. Superimposed to the cortical models, the colored spheres (as in Fig. 1) represent the center of gravity of significant CLs Mid‐Gamma clusters. Labels represent electrostimulation mapping (H, Hand; F, Fingers, 1‐2‐3‐4‐5 individual digits, s/m sensory/motor). Stars indicate electrodes with a late lateral response described below.

In order to evaluate Mid‐Gamma activations for each digit, the tridimensional location of electrodes belonging to the most significant cluster were weighted by their absolute power and represented by the colored spheres on the cortical models in Figure 4.

Across subjects, the vibrotactile task led to clustered localizations of the contralateral fingers, spanning less than 20 mm. For six of the seven subjects, the estimated digital areas were located posteriorly or above the central sulcus, in agreement with anatomical expectations, including for Sub3, who was asleep during the entire study.

The latero‐medial order of the fingers was not fully consistent across subjects, which could be explained by intersubject variability in digital somatotopy (Schweizer et al. 2008).

In comparison, electrical stimulation led to broad representations of the hand/digits and seldom located sensory areas (three subjects).

### Late lateral responses

Four of the seven subjects (1, 5, 6, and 7) underwent a sensorimotor task requiring a finger press in response to the vibrotactile stimuli.

**Figure 5:**
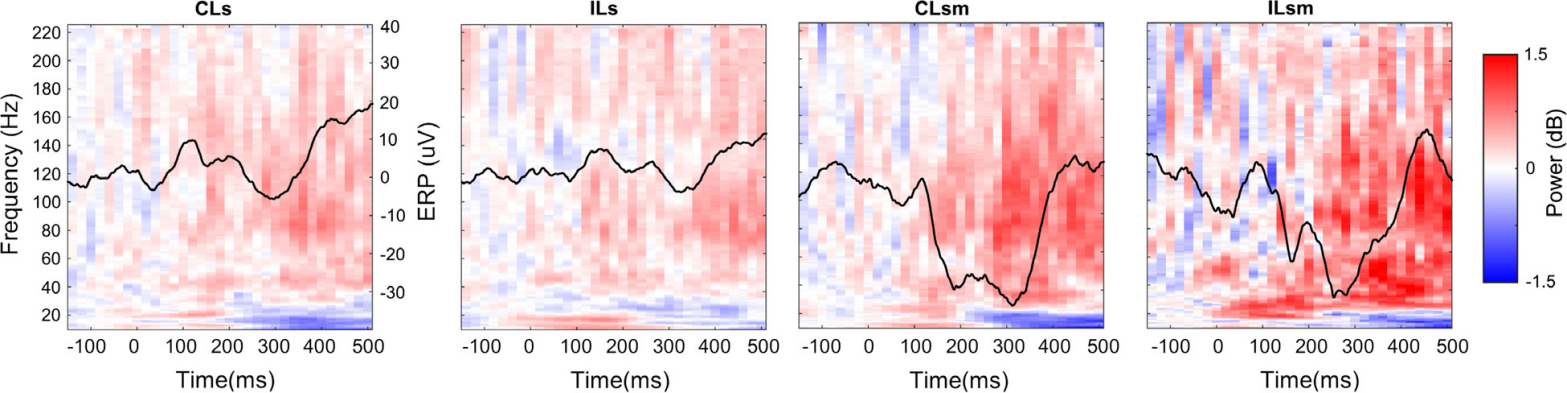
Late lateral response averaged over four subjects.

For those subjects, we observed an additional broadband frequency response associated with two late negative ERP peaks (Fig. 5). These bilateral responses, localized in the electrodes marked in Figure 4 with a yellow star, were stronger under the sensorimotor task than in the sensory only task. Their near‐Sylvian location and the delay to the initial response are relatively consistent with the secondary somatosensory areas and their delayed activation seems to imply a secondary processing or motor planning.

## Discussion

Traditional sensorimotor mapping using electrical stimulation of cortical areas as part of the routine evaluation of patients with medically intractable epilepsy relies on “unnatural” exogenous stimulation through two adjoining electrodes, leads to discomfort, nausea or seizures that often delay the mapping from several hours to the next day, prolonging the process when expediency and defined eloquent areas are needed for surgical decision making. Presently, the accuracy of localization of functional areas is dependent on the subjective assessment of induced sensory or motor responses from a patient and the assessor. For several subjects, standard electrical stimulation in the precentral cortex led to motor responses (Sub5, Sub7, see Fig. 4), which is consistent with previous findings (Penfield and Boldrey 1937; Nii et al. 1996; Haseeb et al. 2007; Fukuda et al. 2008). In our study, the center of gravity of the digital sensory clusters was predominantly located on the central sulcus or in the postcentral gyrus (Fig. 4). This could be explained by the fact that the initial and strongest sensory response was found to be precentral or central (Fukuda et al. 2008), weighting the centers of gravity toward these locations.

In contrast to standard electrical stimulation, this study suggests that vibrotactile mapping is a fast process involving endogenous cortical activity, which (1) can be implemented in real time, (2) offers detection at the single‐electrode level, and (3) elicits clusters of significant activation originating in the precentral cortex or above the central sulcus. The vibrating pad used in this study allowed single digit stimulation and elicited reliable ERP, ERD and/or ERS responses in all subjects, independently of subject wakefulness and mental capacity.

Evoked response potentials were often degraded by interictal background noise and jitter, while the time‐frequency representations showed more consistency across trials and subjects. The use of the clustering‐based statistical approach, taking into account adjacency relationships in time, frequency, and space, led to a reliable detection of significant ERS/ERD clusters in relevant areas within the Mid‐Gamma range.

Across our subjects, Mid‐Gamma ERS clusters arose in areas consistent with anatomical structures, with six subjects presenting a full somatotopic organization of at least four fingers. The remaining subject presented only a single digit on the edge of the grid, hinting that it may not be covering the whole area of interest. The lack of consistent temporal and spatial relationship between ERS and ERD onsets may indicate two distinct cortical processes in the integration of sensory information.

In addition, we observed a late lateral response in areas consistent with SII, and stronger in the sensorimotor task, indicating that this procedure may also be used to localize this area.

The recordings presented in this study were acquired from patients with focal epilepsy undergoing interoperative mapping for surgical focus resection and under the influence of antiepileptic and pain medications. Antiepileptic drugs are believed to reduce cortical excitability and may alter results, although they did not seem to affect this paradigm. Analyses were focused on this target population toward sensorimotor mapping of their individual brains. Hence, ERPs as well as ERD/ERS locations may be strongly affected by their condition and generalization from/to healthy subjects is problematic.

In conclusion, we suggest that vibrotactile stimulation associated with cluster‐based statistics applied on the frequency domain may lead to a more accurate localization of sensorimotor functions than the standard electrical stimulation, provide additional information in the surgical decision making for patients with medically intractable seizures, and assist in predicting functional outcome. Further analysis, applying cluster detection on the full band spectrum, instead of on a per band basis, would lead to cluster localization in the time‐frequency‐space domain instead of time‐band‐space domain and may provide further information on the spectral evolution of elicited activity. Coherence analysis on the single trial level between the medial and the Sylvian‐activated locations may provide further insight on their temporal relationship and whether the latter represent a secondary processing or motor planning stage. This scheme could also be extended to other somatic locations to verify its applicability to full body mapping.

## Conflict of Interest

None declared.
